# The social and behavioral influences (SBI) study: study design and rationale for studying the effects of race and activation on cancer pain management

**DOI:** 10.1186/s12885-017-3564-2

**Published:** 2017-08-25

**Authors:** Cezanne M. Elias, Cleveland G. Shields, Jennifer J. Griggs, Kevin Fiscella, Sharon L. Christ, Joseph Colbert, Stephen G. Henry, Beth G. Hoh, Haslyn E. R. Hunte, Mary Marshall, Supriya Gupta Mohile, Sandy Plumb, Mohamedtaki A. Tejani, Alison Venuti, Ronald M. Epstein

**Affiliations:** 1 Department of Statistics, West Lafayette, Purdue University, Human Development & Family Studies, Indiana, 47906 USA; 2 Purdue University Center for Cancer Research, Regenstrief Center for Healthcare Engineering, Human Development & Family Studies, Fowler Memorial House, 1200 W State Street, West Lafayette, IN 47906 USA; 3 Department of Internal Medicine, Hematology & Oncology Division and Department of Health Management & Policy Ann Arbor, University of Michigan School of Medicine, Ann Arbor, MI 48109-0419 USA; 4 Department of Public Health Sciences, University of Rochester School of Medicine, Family Medicine, Rochester, NY 14642 USA; 5 Biostatistics Department, School of Public Health, University of Michigan, Ann Arbor, MI 48109, 14642 USA; 6 Department of Internal Medicine, University of California Davis School of Medicine, Sacramento, CA, University of Rochester Medical Center, Rochester, NY USA; 7 West Virginia University, Robert C. Byrd Health Sciences Center, Morgantown, West VA 26506 USA; 8 James P Wilmot Cancer Center, University of Rochester Medical Center, Rochester, NY 14642 USA; 9 University of Rochester School of Medicine, Family Medicine, Rochester, NY 14642 USA; 10 Center for Communication and Disparities Research, University of Rochester School of Medicine, Family Medicine, James P Wilmot Cancer Center, Rochester, NY 14642 USA

**Keywords:** Patient-centered communication, Cancer, Racial disparities, Implicit bias, Randomized clinical trial, Field experiment, Standardized patients, End of life care, Palliative care, Pain management

## Abstract

**Background:**

Racial disparities exist in the care provided to advanced cancer patients. This article describes an investigation designed to advance the science of healthcare disparities by isolating the effects of patient race and patient activation on physician behavior using novel standardized patient (SP) methodology.

**Methods/design:**

The Social and Behavioral Influences (SBI) Study is a National Cancer Institute sponsored trial conducted in Western New York State, Northern/Central Indiana, and lower Michigan. The trial uses an incomplete randomized block design, randomizing physicians to see patients who are either black or white and who are “typical” or “activated” (e.g., ask questions, express opinions, ask for clarification, etc.). The study will enroll 91 physicians.

**Discussion:**

The SBI study addresses important gaps in our knowledge about racial disparities and methods to reduce them in patients with advanced cancer by using standardized patient methodology. This study is innovative in aims, design, and methodology and will point the way to interventions that can reduce racial disparities and discrimination and draw links between implicit attitudes and physician behaviors.

**Trial registration:**

https://clinicaltrials.gov/, #NCT01501006, November 30, 2011.

## Background

Racial disparities affect the management of pain for patients with advanced cancer. Compared to whites with advanced cancer, blacks with advanced cancer are prescribed less pain medicine, explaining why black patients with cancer report a greater pain burden than do their white counterparts [1]. Potential contributors to racial disparities in pain management include differences in patient reporting of pain, differences in physician assessment of pain, differences in patient-centeredness of patient-clinician communication [2, 3], and implicit bias [4].

Physicians report that their own ability to perform an adequate assessment of pain is a barrier to successful pain management [5] and that patients have trouble telling them about their pain in general. These problems are accentuated with black patients, who, according to their physicians, do not tend to speak up to tell their oncologists their concerns [6, 7]. Patient race in the context of physician implicit biases affects physician clinical decisions and communication behaviors.

Pain assessment is inherently subjective. It relies on trust in patients’ reports and is influenced by physicians’ implicit stereotypes about black patients. Implicit stereotypes are developed unconsciously through a lifetime of cultural interactions and can surface in the context of the uncertainty surrounding pain and its assessment and steer communication (e.g. word choice or eye contact) and decision-making about pain management in a way that disadvantages black patients [8]. The automatic triggering of these implicit biases is enhanced by cognitive overload due to contextual factors such as complexity of disease, complexity of the psychosocial situation, expressed emotion, racial and cultural differences in language use, and co-morbid conditions. Cognitive overload may be especially relevant for decisions about pain management because pain management involves clinician discretion owing to the absence of objective measures of pain, and paucity of specific clinical practice guidelines.

Stereotypes about black patients are linked to pain management decisions [8]. Black patients are less likely than white patients to have their pain documented in medical records and are less likely to be referred to a pain specialist. Physicians are more likely order urine drug tests for black patients and more likely to refer them to substance abuse treatment [9] despite evidence that black patients are less likely than white patients to use opioids for non-medical purposes [10].

Studies suggest that patients who are more assertive and ask more questions during their visit (“activated patients”) elicit more patient-centered behaviors from physicians, including responsiveness to patients’ concerns and incorporating patients values into decisions [6, 11]. A recent study finds that teaching black patients about pain management and coaching them to discuss their needs for pain relief with their physicians results in improved pain control and elimination of disparities between black and white patients [12]. Such coaching is an example of patient “activation.” Patient activation may mitigate racial disparities by promoting patient-centered communication, yet few studies have examined the effect of patient activation on disparities in pain management.

Our study, the Social and Behavioral Influences (SBI) study, is designed to examine both race and patient activation as factors in pain treatment. The SBI Study is a randomized field experiment that aims to advance the science of healthcare disparities in patients with advanced cancer. This article describes the empirical and theoretical rationale for the study, the rationale for unannounced standardized patient (uSP) methodology, SP role development and fidelity, study measures and study procedures, the analytic approach and potential implications of this research.

### Theoretical framework

**Fig. 1:**
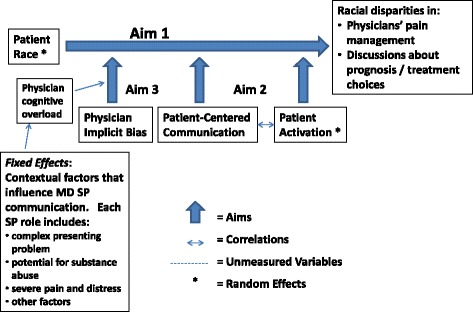
Conceptual model

We examine the effects of patient activation, patient-centered communication, and physician implicit stereotypes on racial disparities in pain management (along with secondary outcomes) applying Street’s ecological model of healthcare communication. An ecological model considers the context of the visit and the larger health care system to model the interaction and mutual influences of patient on physician and physician on patient [13] (See Fig. 1). In addition, we draw from Van Ryn’s model of racial disparities [14]. In this model, disparities in pain assessment and communication are due, in part, to the direct and moderating effects of patient characteristics, physician implicit bias, and contextual factors that occur during clinical conversations. In the SBI study, uSP methodology enables us to examine physician factors in communication and decision-making by manipulating patient factors (race and activation) experimentally while keeping contextual factors fixed.

Standardized patients are actors trained to portray a particular role (or roles) in order to minimize, to the greatest extent possible, inter-patient effects introduced when studying patient-physician communication in real-world clinical settings. [15] In designing the SP roles, we fix contextual factors such as the nature of presenting problems, level of pain and non-verbal expressions of distress. In addition, we accentuate cognitive load by introducing a complex presenting problem, a stigmatized condition (a smoking-related illness), the potential for substance abuse, severe distress, and other factors. Meanwhile, patient activation, as reflected in patients’ communication behaviors, can counteract the effect of physicians’ stereotypes (implicit biases) because it increases physicians’ personal knowledge of patients and prompts physicians to involve patients more actively in care.

Based on this framework, we predict that patient activation will foster individualization of the care that physicians provide and diminution of racial stereotypes as the physicians become more aware of the patients’ (SPs’) concerns and values. Theoretically, physicians who see patients as individuals rather than merely members of a particular group would engage in patient-centered communication behaviors (e.g., eliciting patients’ concerns, providing information, being empathic, and responding to questions). Physicians’ patient-centered communication behaviors may potentially establish a virtuous cycle by reinforcing patient activation by encouraging question asking and participation in decisions.

### Current investigation

Building upon the ecological framework, this study will provide one of the most rigorous tests to date of the effect of both race and patient activation in pain treatment disparities. This study addresses the limitations of observational studies and written and video vignette studies by employing a randomized experimental design to examine observed differences in patient physician interaction by race and patient behavior, while examining moderators including physician implicit attitudes. Our hypotheses are:

1. Physicians’ pain management decisions in advanced care patients will differ between black and white SPs portraying identical roles. Specifically, we hypothesize that black SPs will receive less intensive pain management, i.e. lower total doses of opioids, shorter supply of opioids, and less adequate dosing.
2. Physicians’ communication will differ between black and white SPs. Specifically, we hypothesize that clinicians will ask the black SP fewer pain questions and perform a less thorough assessment of the SP’s pain.
3. Physician differences in behavior by the race of the SP will be attenuated among SPs portraying the activated role. Patient activation will mitigate racial differences in communication behaviors and pain management decisions.
4. Implicit bias will moderate clinician prescribing and communication behavior by race.

## Methods

### Overview

The investigators aimed to recruit individual physicians, primary care and cancer clinics, and health care systems via email and telephone, up to 110 primary care physicians and oncologists from Western NY State, Central/ Northern Indiana and lower Michigan to participate who provide consent to participate in an unannounced SP study of “behavioral and social influences” on health care. With attrition, our recruitment goal is 90 physicians, accounting for inability to schedule visits, physician retirements; 90 physicians will provide adequate power to test our hypotheses. At each site, we will hire and train four SPs (2 black and 2 white and two activated and two “typical”) for whom we will schedule clinic visits with participating physicians. At the time of the visit, physicians will not know that the “patient” is, in fact, an SP. Using covert audio recordings of the visits and SP ratings, we will assess physician communication and prescribing behavior in the management of severe cancer-related pain. We chose to use only male SPs because evidence suggests that racial bias against black men is stronger than that against black women [16, 17], and thus our study aims would have less risk of being under-powered. The protocol calls for each physician to see two patients of the same race because of we did not want to identify any individual physicians might be identified as providing differential care based on race.

The study procedures are separated into four steps. Before the study begins, we pilot each step of the study across all three sites. The Western New York site was slated to begin recruiting physicians and deploying SPs during years 1 and 2, followed by the Indiana site in years 2 and 3 and Michigan in years 3–5. Physicians recruited to the study complete a consent form for participation, a physician questionnaire; they also identify an office liaison to work with the study coordinators at each site to help with scheduling, medical records and canceling tests and follow-up appointments. At least 4 months after recruitment, the first of 2 SP visits is conducted, followed by the second visit at least 4 months later (see Visit Procedures for additional details). Approximately 2 months after the physician sees the second SP, we send the physician an email or fax asking whether they suspect that they have seen an SP; then the study team requests a copy of the SP’s record and study physicians complete an online Implicit Association Test designed specifically for the study.

### Selection of study sites

We chose to recruit physicians from three geographic regions with corresponding differences in communities, healthcare systems, and local practice culture. The Western New York region is a mix of urban and suburban sites with a substantial population of African-Americans and Latinos, a broad socioeconomic mix and a mixture of University-based and private oncology practices and community-based primary care practices. The North-Central Indiana region is in the heart of rural Indiana with a mixture of large health care organizations and community based practices that are being integrated into larger healthcare systems. The Michigan oncology practices are community-based; the primary care physicians are recruited from two large health systems.

### Eligibility, recruitment, consent

We obtained IRB approval from each of the corresponding academic institutions and medical systems prior to physician recruitment. The study is designated as a deception study at the University of Michigan but not at the two other sites. Participating physicians sign written, informed consent. Complete inclusion and exclusion criteria are presented in Table 1, and participant eligibility is verified before consent.

**Table 1:** Inclusion and exclusion criteria for oncologists and primary care physicians

Participant	Inclusion criteria	Exclusion criteria
Oncologist	Oncologists that care for patients with solid tumors and who would likely see a patient with lung cancer Not planning to leave the practice or retire within the next year	Non-physicians, Oncologists who exclusively care for patients with hematologic malignancies, those who specialize in exclusively genitourinary, breast, hematologic and neurologic cancers.
Primary Care	Not planning to leave the practice or retire within the next year	Non-physicians

At each site, we recruit medical oncologists who care for patients with solid (non-hematologic) cancers and primary care physicians (family medicine physicians and internists) using email, telephone and in-person meetings. We deploy SP visits to oncologists first, in order to avoid primary care referrals to oncologists who could potentially be scheduled to see SPs in the same or neighboring networks, hospitals, or physician practices. Interested physicians meet with the study personnel (site PIs, study coordinator, and/or research assistant) to learn about the project, provide written consent, and complete the baseline surveys. The consent document seeks each oncologist’s agreement to 1) complete initial baseline questionnaires, 2 & 3) complete two patient visits with unannounced SPs, during the next 18–24 months after consent, that are covertly audio-recorded, 4) a standardized patient detection form and the Pain Implicit Attitudes Test, a modified version of the well-known IAT, [18], at least a month after the two visits. Physicians are told that the SBI study “examines social and personal factors that affect clinical care and outcomes” and that these factors might include “patient age, gender, race/ethnicity, income, education, communication style, disease, symptoms, and functioning, as well as physician factors.” Further, we inform physician participants that the study would “identify communication behaviors that improve mutual understanding between patients and physicians.” Once physicians agree to participate, we ask them to provide the name of an office liaison. The consent document describes the four steps of the study. Participating physicians receive $600 for completing all steps of the study ($150 for each step completed).

Because of increased scrutiny of patient identification, we work closely with practice managers to establish what would qualify as an acceptable photo ID (such as a work badge) or create work-arounds so that patients would not have to show a photo ID at all; clearly, creating false state or federal identification cards would not be permissible. Similarly, we create false callback numbers for patients and devise methods for checking phone mail and responding accordingly.

### Standardized patient roles

We hire and train two sets of white SPs and two sets of black SPs at each site. The four SP roles at each site include 1) a black individual who portrays an activated patient, 2) a black individual who portrays a typical patient, 3) a white individual who portrays an activated patient, and 4) a white individual who portrays a typical patient. Both black and white activated and typical pairs portray them identically. In order to further standardize the role and where possible, actors are matched on physical appearance and interpersonal style when assigning them to the typical versus active SP role. SPs report at each visit that they were treated for lung cancer in another state and that they moved to be closer to one of their adult children, explaining their need to find a new physician. The SPs report bony pain, rated 7 out of 10, not relieved by current medication such that they have been taking more than the prescribed doses due to escalating pain. For the past 2 weeks, SPs report taking 2 tablets of hydrocodone 5 mg /acetaminophen 500 mg (Lortab® or Vicodin®) every 3 h instead of 2 every 4 h as prescribed. The four SP roles created are identical except for two factors – SPs’ race and patient activation, as shown in Table 2.

**Table 2:** SP Characteristics by Race and Activation Level

Standardized Patient Race	Activation Level: High	Activation Level: Low	Total N of SPs
Black	1	1	2
White	1	1	2
Total N	2	2	4

Activation is operationalized in this study according to behavioral criteria developed by Street et al., Hibbard et al., and Kaplan et al. [19–22]. Activated SPs ask more direct questions about their pain management, their prognosis, and the risks and benefits of pain medications. They request information, ask questions when they do not understand, and redirect the discussion when their concerns are not addressed. (see Table 3) In addition, activated SPs are trained to bring a list of questions, express at least one concern about side effects of treatment and or prescription modifications, and interrupt the physician at least once to ask for more information. If the physician has already provided the answer to an activated SP question before the SP asks it, the activated SP has been trained to use supplemental questions that invite further clarification from the physician in order to make sure that an activated SP role is portrayed. Using a series of pilot visits, activated patients’ role presentations are calibrated so they do not appear demanding or question the physician’s competence. Typical patients are not as engaged in care, as evidenced by questions only about how to follow through with treatment, relatively few emotional concerns expressed, general satisfaction with information presented, and indicating understanding without asking follow up questions.

**Table 3:** Sample Activated SP Questions & Comments

1. I am wondering if I should be taking more pain medication – should I?
2. You know, the pain seems to be getting more bothersome. Does the pain medication stop working after a while?
3. Am I going to get addicted to the medication?
4. I know things are not good, but can you be realistic about what’s the best case scenario and what’s the worst case?
5. What are my options at this point? You know, I really prefer to be comfortable at this point.

### Standardized patient training

Each site is responsible for hiring SPs. SPs receive 50 h of training in the role before deployment, including a 3-day intensive training at the University of Rochester, Purdue University, or the University of Michigan with the PIs and the trainers from all 3 sites. During training, roles are piloted at all sites with clinicians who are unaware of the study hypotheses in order to achieve roles sufficiently distinct, credible, unlikely to raise suspicions that the patient is an SP, and unlikely to introduce other confounding concerns (e.g., mental illness).

For this study, we build on the clinical biography and extensive script developed for the pilot study. [23] The detailed script, which we use to standardize training at all three sites, describes likely physician questions and appropriate SPs responses to physician questions. During the first months of this project, we review and update both roles and ask for feedback from local experts at each site to ensure that the roles are psychologically and medically believable. Training focuses on learning the biographical details and portraying the attitudes and emotions of the role.

SPs are monitored throughout the study to maintain 90% or higher role fidelity using a fidelity rating scale. The scale includes content items that assess the accuracy of the facts presented as well as rating scales to calibrate tone of voice, level of emotionality portrayed, and non-verbal pain behaviors. In addition, SPs receive active training during the time visits are taking place. Activated SPs are trained separately from the typical SPs, and the roles are not at any time shared or discussed with SPs portraying the other role. All SPs are blinded to the study hypotheses and are not told that activation or race are related to the study hypotheses. To monitor fidelity and offer ongoing feedback during data collection, SP trainers listen to audio-recordings within 2 business days of each visit for the first 15 visits, after every third visit thereafter, and more frequently if needed for feedback to the SPs.

### Randomization of SPs

We employ an incomplete randomized block design in which each block does not receive all treatments. Each physician is visited by 2 *different* SPs, both of the same race, but differing according to activation. Each physician experiences 2 of the 4 possible SP roles – either a) black activated and black typical (non-activated), or b) white activated and white typical; the order of presentation of the SPs is randomized, such that half of the physicians see the activated SP first, and the other half see the typical SP first. Visits are at least 4 months apart.

### Visit and standardized patient procedures

We arrange all visits through the office liaison in order to manage anticipated problems such as insurance verification and identity checks. The office liaison agrees to arrange “detours” around usual administrative details that office staff members might raise. We stress to the office liaison about the importance of not disclosing the identity of the SP to anyone in the office who might inform the physician. If the practice has been closed to new patients, the study coordinator works with the office liaison to devise a plausible excuse (e.g., stating that the SP is the relative of a current patient) to include the patient on the physician’s office schedule.

Before each physician visit, the SP meets with the SP trainer or study coordinator for a pre-visit meeting to go over details about the role and obtain necessary documents (ID card, insurance card, medication lists, recent labs, etc.) and recorders. SPs also review office logistics and new patient handouts if available. About 1 week prior to the visit, participating physicians are sent mock medical records, with fake contact information for physicians and clinics. If physicians follow up on the contact information, they would encounter convincingly designed mock websites, and phone numbers with phone trees that ultimately ask the caller to leave a voice mail.

On arrival to the office, SPs activate audio recorders and present (fake) photo IDs and/or insurance cards. In some cases, office liaisons advise the study coordinators to avoid using insurance cards and to develop story lines about self-pay or billing out-of-state insurance after the visit due to concern about tipping off office staff or the physician about SP identity. The office staff registers the SP and creates an electronic or paper medical record as if the SP’s were a real patient visit. The SP role includes cooperation with all aspects of the visit, but the SP declines any invasive medical procedures (e.g., blood draws) and any radiographic studies; they also decline any oral, topical or injectable medications that might be offered during the visit. SPs carry fabricated reports of recent laboratory studies to avert blood draws during the visit.

Immediately following the visit, study coordinators void any prescriptions using a rubber stamp “VOID,” complete an SP Post-Visit Reporting Form assessing specific elements of history taking, physical examination, and medical decision-making, and meet with the study coordinator to return all materials (audio-recorder, prescriptions, questionnaire, ID cards, after visit summaries, lab requisitions, billing information and post visit measures). They debrief with the SP trainer after each visit about how the SPs thinks the visit went, if there were any logistical problems, any difficulties encountered in the visit, or any problems portraying the role.

We intercept electronic prescriptions by calling the pharmacy to cancel them. Either the SP or a research team member cancels all scheduled lab tests and follow-up appointments. Some offices will not release the prescriptions after the SP completes the physician visit so we make arrangements to leave them with office liaisons at checkout. Office staff are notified that the SP would not be returning (various alibis are provided – e.g., that patient is going to move in with a relative in another part of the state, chose another physician, etc.), and the office is instructed to cancel any follow-up appointments, procedures, or case conferences. Physician office staff treats records (electronic or paper) as they would for a real patient who would not be returning to the office. Approximately 2 months after the physician sees both SPs, we send the physician an email or fax asking whether they suspect that they had seen an SP. After seeing two SPs and completing the detection information, the study team requests a copy of the SP’s record. The Pain IAT is administered immediately afterwards. Physicians are given the option to complete the IAT on a personal computer or have the research assistant bring a computer to the office to complete the IAT. We describe these instruments in more detail below.

### Outcome measures

#### Pain medication prescribing

The primary outcome for this study is physicians’ management of patients’ cancer pain. First, we calculate from prescriptions the total daily prescribed dose of each medication and, for opioids, total daily morphine equivalent using standardized opioid conversion charts. When medications are written “prn” or “as needed,” our calculations assume that all doses would be taken. We also calculate the total number of doses dispensed. We also made note of non-opioid medications prescribed, although these were not related to the primary study hypotheses.

### Physician-standardized patient communication

The audio-recorded office visits are coded using behavioral coding systems for Pain Assessment, Prognosis and Treatment Choice Communication, and Eliciting and Validating Concerns [24]. The Measure of Pain Assessment (MPPA) examines the degree to which physicians assess patient pain based on items used in self-report questionnaires. [25–28] and assessments of patient-clinician communication [29–33] The instrument is used in the pilot study to measure the thoroughness of physicians’ assessment of patients’ pain. Examples of items are “onset” (when start and duration), “location,” and “intensity/severity.” We will assess prognosis and treatment choice communication from the audio-recordings using the PTCC, which we developed in our pilot and recently used in a large randomized intervention trial to improve communication in advanced cancer [34]. These items assess physicians’ communication of diagnostic and prognostic information and the treatment options that may be offered to advanced cancer patients. Sample items are “Physician asks if patient wants to know more about his or diagnosis” and “Assessing if patients understand their diagnosis.” These items are coded using the same physician response scale used to measure the depth of pain assessment (MPPA). See Tables 4 & 5 for PTCC and MPPA items.

**Table 4:** PTCC Items

Items
1. Cancer Knowledge: Assessing patient’s knowledge of state of disease
2. Open Door: Asking if the patient wants to know about the prognosis, survival, curability/the future or indicating common questions that people have about the prognosis, survival, curability, future quality of life, or palliative care.
3. Understand Prognosis: Assessing the patients’ understanding of their prognosis.
4. Changing for the Worse: Discussion of how the disease trajectory is changing for the worse.
5. Quality of Life: Discussion of quality of life in the future
6. Palliative Care: Discussing palliative care treatment
7. Advanced Directives: Discussing advanced directives
8. Curability: Discussing if the cancer can be cured.
9. Survival Time: Discussing estimates of survival time.
10. Best Worst Case: Discussing best case and worst case scenario
11. Double Frame: Double Framing Survival/Curability Estimates

**Table 5:** Measure of Physician Pain Assessment Items

Physician Discussing or Asking about	
1. Acknowledging pain	
2. Onset, duration, temporal	
3. Location	
4. Aggravating /alleviating factors	
5. Pain Origins	
6. Interference	
7. Description of Pain	
8. Rate pain on **0**–**10** scale	
9. Physician Role in Pain Management	

#### Physician survey measures

The physician measures completed at the time of enrollment and consent are outlined in Table 6. Physician demographics include age, gender, race, specialty and training information (i.e., board certification, fellowships completed) and practice information (number of patients seen per week, make-up of patient population, ownership of practice, type of provider plans practice participates in, use of an EHR, practice location, etc.) Physician burnout is measured using the emotional exhaustion subscale from the Maslach Burnout Inventory (MBI) [35, 36]. The MBI is widely used and validated with healthcare personnel. Physician empathy was measured using the perspective-taking subscale of the Jefferson Scale of Physician Empathy (JSPE)-A Empathy. The JPE is based on a cognitive definition of empathy (e.g., the physician understands the patient’s experience) and reports good reliability [37].

**Table 6:** Schedule of Measures completed by physicians, Coders, & Standardized Patients

Domain	Measure	Study entry	Post Visit 1	Post Visit 2	2–4 month Follow up
Physician Questionnaires / Measures					
Demographics	Age, gender, race, specialty, practice information, etc.	x			
Physician Burnout	Maslach Burnout Inventory (MBI)	x			
Physician Empathy	Jefferson Scale of Physician Empathy (JSPE)- Perspective Taking Subscale	x			
Psychosocial Aspects of Physician Care	Physician Belief Scale (PBS)– Burden Subscale	x			
Mindfulness	Observing and non-reactivity subscales	x			
Attachment	RQ Attachment Scale	x			
Need for Closure	Need for Closure Scale (NFC)	x			
Comfort Prescribing Pain Medication	PAROPM (developed for this study)	x			
After Visit Physician Measures					
Prescriptions	Extracted from prescriptions given to SPs		x	x	
Detection Fax	Sent to physician 3 weeks after last SP visit				X
IAT	Pain Implicit Associations Test				x
SP Questionnaires					
Measures	SP perception of patient empathy, satisfaction with overall care, quality of pain discussion, quality of prognosis discussion, physician nonverbal, Rochester Physician Communication Scale		x	x	
Coding of Transcripts from Audio Recordings					
Pain Coding	Measure of Physician Pain Assessment		X	x	x
Prognosis Coding	Prognostic and Treatment Choices (PTCC)		x	x	x
Shared Decision Making	SDM Coding Scale [44]		x	x	x
Eliciting and Validating Patient Concerns	Exploring and Validating Patient Concerns		x	x	x

Physicians’ beliefs about psychosocial aspects of patient care are assessed using the Physicians’ Beliefs about What Patients Want 6-item subscale of the Physician Belief Scale. Higher scores reflect physicians’ belief that patients’ psychosocial issues are a part of a physician’s role [38, 39]. We developed a 3-item scale asking about comfort with prescribing opioids. Items include, “In general, I am more reluctant to prescribe opioids for pain than my colleagues are.” We used the Baer Mindfulness scale to measure two facets of mindfulness - observing (8 items) and non-reactivity (7 items) [40]. The Relationship Questionnaire assesses adult attachment styles [41]. The Need for Closure (NFC) scale assesses the tendency to rely on cognitive biases when making decisions, and correlates with racial biases. The NFC moderates the association between intergroup contact and negative racial attitudes [42]. At least 1 month after completing the second SP visit, physicians complete the Implicit Associations Test (IAT), adapted for this study from prior validated versions of the IAT. The IAT measures implicit biases using automatic association tests to assess how the brain links concepts. While the classic race IAT [18] has been widely used, we developed a healthcare focused IAT more relevant to assess implicit biases in physicians. Adding the task of recognizing pain to the classic race IAT creates a measure examining implicit bias in regards to race and pain management.

#### Standardized patient questionnaire

After completing each visit, SPs are asked to rate their satisfaction with overall care, quality of pain discussion, and quality of prognosis discussion on a 6-point Likert scale. SPs are asked whether the physician prescribed pain medication at the visit and their perception of how reluctant or enthusiastic the physician was about prescribing the medication using a 5-point Likert scale.

SPs also answer questions from the Jefferson Scale of Patient Perceptions of Physician Empathy (JSPPPE) [43]. The JSPPPE is a brief five-item scale developed for measuring patient perceptions of their physician’s empathy. Patients respond using a 7-point Likert scale (1- strongly disagree, 7 – strong agree).

SPs report nonverbal communication using a measure developed for the study. The measure consists of seven questions scored on a five point Likert scare (1 = poor, 5 = excellent). Items include “the physician maintained appropriate eye contact with me;” “looked at me instead of a computer/laptop/tablet screen/charts;” and “gestured/nodded their head at me when appropriate.” The study team trains all SPs regarding definitions of nonverbal communication to increase reliability of reporting nonverbal behaviors.

SPs assesses physician’s communication skills using the Rochester Communication Rating Scale, [44] a 19-item scale developed to assess patient-centered communication skills of physicians. Components of patient centered care that are assessed from the patients perspective include eliciting the patient’s perspective, understanding the psychosocial context, developing a collaborative relationship and activity involving the patient in decisions about his or her health.

We use a Role Fidelity Form to assess SPs accuracy to the role. Portrayals are calibrated during the 30 h of extensive training to achieve greater than 90% role accuracy on a 100–point role fidelity scale that includes measures of verbal content and emotional valence of the role.

#### Sample size determination

Our sample size goal is 91 physicians, assuming that 158 visits will have been conducted in total, accounting for attrition. Statistical power is estimated for the fixed effects corresponding to primary study hypotheses using a mixed-effects model with a random intercept capturing between physician variance. We assume moderate nesting of outcomes within physician (intra-physician correlation coefficient (ICC) of 0.3 to 0.5). The effective sample sizes resulting from 158 repeated observations and ICC = 0.03 are 122 for a physician level effect and 226 for a within-physician effect, e.g., activation and activation-by-SP race interaction effects [45]. The effective sample sizes resulting from 158 repeated observations and ICC = 0.05 are 105 for a physician level effect and 316 for a within-physician effect. Given this, a physician effect equivalent to a standardized regression coefficient of 0.25 and 0.27 can be detected with power of 0.8 for ICC of 0.3 and 0.5, respectively. A within-physician effect equivalent to a standardized regression coefficient of 0.16 and 0.19 can be detected with power of 0.8 for ICC of 0.5 and 0.3, respectively. Outcomes with higher levels of nesting will result in less statistical power at the physician level and more statistical power at the within-physician level.

#### Planned analytic approach

Our primary analytic method will be generalized mixed-effects regression modeling using a random intercept to adjust for within physician nesting of outcomes. In these models, associations between predictor variables and study outcomes will be estimated with fixed-effect regression coefficients.

## Discussion

The SBI study addresses important gaps in our knowledge about racial disparities in pain management between white and black patients with pain due to advanced cancer and potential effects on physician behavior of patient activation. It also provides information about potential mechanisms of these disparities, including physician demographics, explicit physician attitudes (e.g., towards opioid prescribing and patient-centered care), physician psychological attributes (e.g. mindfulness and need for certainty), and physicians’ implicit associations regarding race and pain management (measured using the IAT). We use novel standardized patient methodology to control variability in patient presentation. This study is innovative in aims, design, and methodology and will point the way to interventions that can reduce racial disparities and discrimination and draw links between implicit attitudes and physician behaviors that have not yet been investigated.

We have addressed several threats to external validity of the study. By triangulating three different operationalizations of activation, we produce a role that aggregates all of them, presented in moderation so that the activated role is credible and not seen by physicians as aggressive or demanding. We create plausible false medical records that are reviewed by oncologists and primary care physicians for authenticity, and were rarely questioned when deployed in our pilot work. We create credible false websites and contact information for the purported physician and clinic where the standardized patient claimed to have received care, including phone mail. The attention to these kinds of details makes an otherwise difficult study credible.

We have overcome several logistical challenges to implementing the SBI study. In prior SP studies, physicians could be approached directly to gauge their interest in participating, with few exceptions. In the current study, we have to go to large integrated health systems and address the concerns of clinical directors responsible for up to 300 potential physicians, especially their concerns regarding the impact of the study on work flow and billing. Thus, refusal by one administrator could potentially disqualify hundreds of physicians. Nonetheless, we will have achieved recruitment targets that provide adequate power for analyses; the physician sample is diverse and reflects the physician population in those regions.

Even if we fail to confirm the main study hypotheses, the study will provide a rich dataset for examining secondary hypotheses that link physician self-ratings and their observed behavior. For example, the degree to which physicians have insight into their own communication behaviors is not clear and we can find answers by triangulating self-report and audio-recorded data about communication style. Failure to identify racial disparities in prescribing can identify the degree to which pain prescribing in cancer is seen as discretionary (and thus more subject to bias), and may stand in contrast to prescribing for non-cancer conditions, such as chronic low back pain, in which the majority of pain disparities research has been conducted. We have an opportunity to determine whether activation has differential effects on communication depending on whether the patient is black or white.

Finally, we have the opportunity to investigate in greater depth systems issues in providing care to patients in pain and those with advanced cancer. By charting their journey through the health care systems – tests, referrals, prescriptions, and other patient instructions – we can identify important gaps in care.
